# Nanoparticles of Liquid Smoke Rice Husk Inhibit *Porphyromonas gingivalis*

**DOI:** 10.1055/s-0042-1749154

**Published:** 2022-07-12

**Authors:** Ira Arundina, Indeswati Diyatri, Wisnu Setiari Juliastuti, Theresia Indah Budhy, Meircurius Dwi Condro Surboyo, Benni Iskandar, Anisa Nur Halimah, Azzahra Salsabila Adira Moelyanto, Sheryn Marcha Ramaniasari, Gustiadi Saputra

**Affiliations:** 1 Department of Oral Biology, Faculty of Dental Medicine, Universitas Airlangga, Surabaya, Indonesia; 2 Department of Oral Pathology and Maxillofacial, Faculty of Dental Medicine, Universitas Airlangga, Surabaya, Indonesia; 3 Department of Oral Medicine, Faculty of Dental Medicine, Universitas Airlangga, Surabaya, Indonesia; 4 School of Pharmacy, College of Pharmacy, Taipei Medical University, Taipei, Taiwan; 5 Sekolah Tinggi Ilmu Farmasi, Pekanbaru, Riau, Indonesia; 6 Vocational Degree, Universitas Airlangga, Surabaya, Indonesia; 7 Bachelor Dental Science Program, Faculty of Dental Medicine, Universitas Airlangga, Surabaya, Indonesia; 8 Magister of Immunology, Faculty of Medicine, Universitas Airlangga, Surabaya, Indonesia

**Keywords:** liquid smoke, rice husk, nanoparticle, antimicrobial, *Porphyromonas gingivalis*

## Abstract

**Objective** Utilization of liquid smoke rice husk can be used as an alternative treatment because of the antimicrobial properties. Advances in drug delivery systems are increasingly developing to increase the bioavailability of drugs and reduce the side effects of these drugs, namely nanoparticles. In this study, nanoparticles of liquid smoke rice husk (nLSRH) were tested the antimicrobial against *Porphyromonas gingivalis* .

**Materials and Method** This type of research is an experimental *in vitro* laboratory using *Porphyromonas gingivalis* culture. nLSRH contained liquid smoke rice husk concentration of 1, 2.5, 5, 7.5, 10, 12.5, 15, and 17.5%. The antibacterial was performed using the dilution methods.

**Results** The nLRSH concentration of 1% showed clearest medium. The highest number of colonies *Porphyromonas gingivalis* was observed at nLSRH concentration of 1% (40.3 colony-forming unit [CFU]) and decreased at a concentration of 2.5% (11.3 CFU); other concentration or no bacterial colony growth was found. The nLSRH concentration of 2.5% can be determined as the minimum inhibitory concentration and nLSRH concentration of 5% can be determined as the minimum bactericidal concentration.

**Conclusion** nLSRH have antimicrobial activity against *Porphyromonas gingivalis* . This finding able to drive the next research to develop nLSRH as gingival and periodontitis disease is caused by *Porphyromonas gingivalis.*

## Introduction

Periodontitis is an inflammation of the periodontal tissue that results in damage to the alveolar bone, besides that there is pocket formation indicating that there is a pathological deepening of the gingival sulcus. This causes the loss of an *attachment loss* and tooth loss in adults. 1 Periodontitis that often occurs is chronic periodontitis, often due to colonization by *Porphyromonas gingivalis* with a prevalence rate of ∼80.5% cases. 2 Periodontitis treatment performed includes controlling the accumulation of plaque in the oral cavity and also root canal treatment to eliminate bacteria that cause infection 3 Administration of antibiotics is used as a support in reducing the number of colonies of *Porphyromonas gingivalis,* but it should be noted that antibiotics can cause side effects that can reduce drug concentrations in target cells and a resistance to bacteria due to improper use and not as recommended, it will cause bacteria to grow, easily adaptable and immune to antibiotics. 4

The latest research is growing related to the processing of natural ingredients as a suitable alternative as an antimicrobial that is safer for consumption in the long term. 5 One of the developments of liquid smoke rice husks the health sector is as an antimicrobial agent; this is supported by the stimulation of a healing process in the oral mucosa against several bacteria. 6 7 The active content of liquid smoke rice husks forms origin of cellulose, hemicellulose, and lignin. This component through the pyrolysis process produces chemical compounds such as phenol, guaiacol, and acetic acid. 7 The content of phenolic compounds in liquid smoke rice husks can work more effectively toward more specific target cells from a pharmacological aspect developed through the latest *novel drug delivery system* advancements. A oral drug delivery system that is able to regulate pharmacokinetic capabilities by reducing the toxicity of a drug and increasing drug effectiveness on target cells. 8 It has a nanometer size, which is around 1 to 100 nm, in the form of particles that are dispersed and encapsulated with polymers to form a nanoparticle matrix. 9 10 11 By that reasons, the widespread disadvantages of using antibiotics and the development of advances in drug delivery systems are the driving factors for determining the antimicrobial activity of nanoparticles liquid smoke rice husks (nLSRH) to *Porphyromonas gingivalis* .

## Materials and Methods

### Liquid Smoke Rice Husks

The liquid smoke of rice husk used in this study was obtained with pyrolysis process from 1,760 g of rice hull that was air-dried at room temperature as previous study. 12 Liquid smoke of rice husk was diluted by sterile water to make concentration of 1, 2.5, 5, 7.5, 10, 12.5, 15, and 17.5%.

### Nanoparticles of Liquid Smoke Rice Husks

Each concentration of liquid smoke rice husk (1, 2.5, 5, 7.5, 10, 12.5, 15, and 17.5%) was made as nanoparticles with chitosan (Bio-chitosan, Indonesia) and maltodextrin (Qing Huadong Lihua Starch, China). Chitosan (1.5% w/v) and maltodextrin (8.5% w/v) are dispersed in a solution of glacial acetic acid water (1.0% v/v). The chitosan–maltodextrin nanoparticles are made by complexation of chitosan polyelectrolyte with maltodextrin and additional chitosan ionic glass with sodium tripolyphosphate anion. Chitosan and maltodextrin are dissolved in liquid smoke rice husks. Sodium tripolyphosphate (1.0 mg/mL) is added to the mixture and stirred using a magnetic stirrer at 200 rpm for 30 minutes at room temperature. The nanoparticles are isolated by centrifugation at a speed of 3,000 rpm in a 50 mL cone tube for 30 minutes at room temperature. Supernatants is discarded and nanoparticles are filtered in a vacuum using Whatman #2. The nanoparticle solution is heated at 50°C into a water bath for 15 minutes and homogenized using a speed rotor-stator homogenizer at 5,200 rpm for 2.5 minutes. 13

### Characterization of nLSRH

The characterization process was performed based on the Malvern method to determine the particle size (nm) measured using the Zetasizer Nano ZS (Malvern Instrument Ltd, UK) on the sample. Measurement of particle size using dynamic light scattering.

### Preparation of *Porphyromonas gingivalis*

Bacterial stock using *Porphyromonas gingivalis* ATCC 33277 was obtained from the Research Center of the Faculty of Dentistry, Universitas Airlangga, Surabaya.

### Antimicrobial Test

The test was performed using the dilution and diffusion method and analyzed by visualization. Research is a laboratory experiment with eight treatments given at different concentrations with two control groups and repeated three times. In the positive control tube, 0.1 mL of bacteria will be given *Porphyromonas gingivalis* in brain heart infusion broth (BHIB) media. The negative control was 0.1 mL of nLSRH. The treatment group was divided into eight test tubes containing 0.1 mL of bacteria *Porphyromonas gingivalis* Mc. Farland (1.5 × 10 ^8^ CFU/mL) in BHIB media and nLSRH that have been diluted with various concentrations. 7 Measurement of the value of the minimum inhibitory concentration (MIC) can be done visually by analyzed the turbidity of the media and spectrophotometry. 14

The diffusion methods were used to calculate the number of colonies *Porphyromonas gingivalis* on a Mueller Hinton agar using a colony counter. The lowest colonies were assumed as the minimum bactericidal concentration (MBC). 15

## Results

### Characteristics of nLSRH

The characteristic of nLSRH has a lower pH as 3.41. The color bright yellow with average of nanoparticles as 33 days.nm.

### Antimicrobial Test nLSRH to *Porphyromonas gingivalis*

**Fig. 1:**
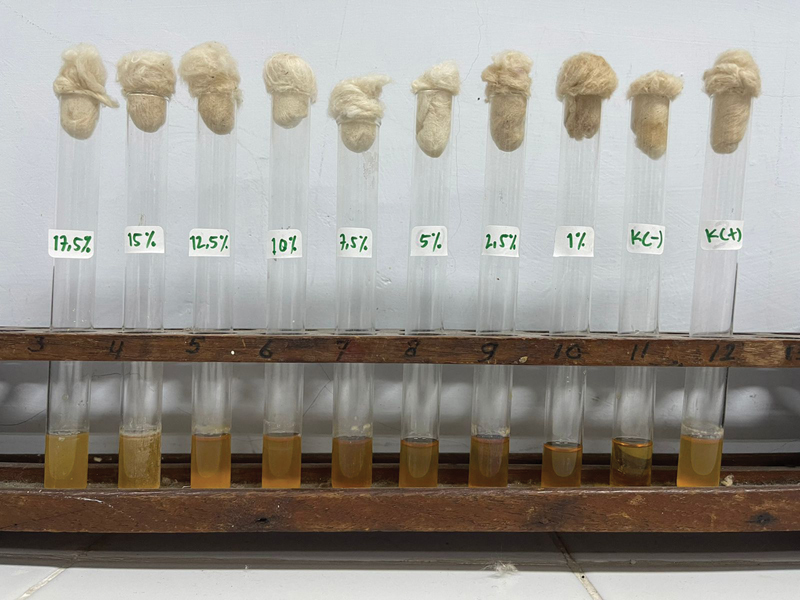
Colony growth of *Porphyromonas gingivalis* on brain heart infusion broth.

**Fig. 2:**
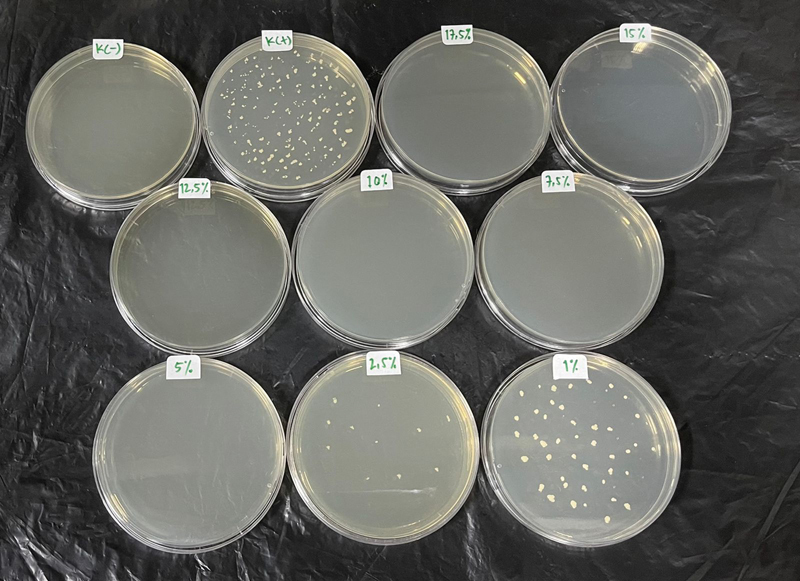
Colony growth of *Porphyromonas gingivalis* on Mueller Hinton agar.

The nLSRH concentration of 1%, liquid media looked clear compared with the control positive media ( Fig. 1 ). The highest number of colonies *Porphyromonas gingivalis* was observed at nLSRH concentration of 1% (40.3 CFU) and decreased at a concentration of 2.5% (11.3 CFU) ( Table 1 ); other concentration or no bacterial colony growth was found ( Fig. 2 ). The nLSRH concentration of 2.5% can be determined as the MIC and nLSRH concentration of 5% can be determined as the MBC.

**Table 1:** The calculation of *Porphyromonas gingivalis* colonies on *Mueller Hinton* agar

**Media**	**Colonies (CFU)**
Control (−)	–
Control (+)	157.7
nLSRH 1%	40.3
nLSRH 2.5%	11.3
nLSRH 5%	–
nLSRH 7.5%	–
nLSRH 10%	–
nLSRH 12.5%	–
nLSRH 15%	–
nLSRH 17.5%	–

Abbreviations: CFU, colony-forming unit; nLSRH, nanoparticles of liquid smoke rice husk.

## Discussion

This research was conducted to predict the antimicrobial activity of nLSRH to *Porphyromonas gingivalis.* The test was performed by determining the MIC and MBC of each concentration of nLSRH. The *Porphyromonas gingivalis* in this study due to its colonization in the oral cavity can cause a periodontitis that can result in conditions such as inside pockets in the gingival sulcus, periodontal tissue inflammation to the damage, and tooth loss in adult cases. 1

The nLSRH concentration of 2.5% was determined as MIC and nLSRH concentration of 5% was determined as MBC. The nLSRH showed better antimicrobial activity compared liquid smoke rice hush in the previous study. 7 The liquid smoke rice husk showed the MIC at the concentration of 10% and MBC at concentration of 12.5% to *Porphyromonas gingivalis* . 7 16 The large decrease in the number of colonies occurred due to the phenolic compounds carried by the nanoparticles working effectively on the target cells. The content of phenolic compounds in nLSRH indicates the presence of molecular activity in it in the form of proton distribution assisted by protonophores across the hydrophobic core of the membrane. When protons enter the membrane, there is an increase in conductance in the phospholipid layer that results in a decreased membrane permeability function. 17 The polar form of protons interacts with phenol to form a weak hydrogen bond that can cause changes in protein structure that have a role in the process of cell metabolism with enzymes in the catalytic process in bacteria and cause an imbalance in the formation of proteins in cells so that bacteria will die slowly. 2 18 19 20

In some antimicrobials that enter the body will be inhibited by the activity of bacterial enzymes and membranes that are difficult to penetrate, such as *Porphyromonas gingivalis* that is a gram-negative bacterium. 21 In nLSRH which contain phenolic compounds that can overcome antimicrobial resistance by interfering with bacterial metabolism through deactivating enzymes due to interactions with protease and pectatelyase enzymes so that enzymatic protein deposition occurs. 22 In addition, the inhibition of the availability of metal ions can have an effect as an indication of damage to the permeability of the bacterial cell membrane with leakage of the cytoplasmic membrane due to an increase in K+ ions outside the cell. Because these ions have an important role in enzymatic activity, ribosome unity, and stabilization of bacterial RNA, so that excessive ionic activity outside the cell makes the bacteria weaker and antibacterial activity will enter and cause the bacterial lysis process to accelerate. 23 24

When nanoparticles begin to enter the body, they interact with gram-negative bacteria, especially in the lipopolysaccharide portion of the bacteria; this interaction will disrupt the activity of the bacterial cell membrane, thereby accelerating the penetration of compounds carried by nanoparticles to their target cells. 25 Selection of nanoparticle components can increase their biocompatibility as is the case with chitosan as a polymer. The negative charge of the bacterial membrane will attract each other due to the electrostatic bonds possessed by chitosan so that it can interfere with the activity of enzymes in bacterial cells. Maltodextrin has an advantage in the stability of the phenol compounds that are brought in, thereby increasing the phenol concentration during the penetration process through the bacterial cytoplasmic membrane. The higher the phenolic compounds carried, the higher their ability to penetrate the membrane and antimicrobial activity will increase with the help of cross-linking that occurs with tripolyphosphate so that bacteria will lysis. 26 27 28

The limitations of this study are limited to antibacterial observations of *Porphyromonas gingivalis.* But the results of this study provide preliminary evidence regarding the potential of nLSRH as a material candidate for gingivitis and periodontitis therapy. Subsequent research needs to be tested to determine the effectiveness and mechanisms that play a definite role in nLSRH as a treatment for gingivitis and periodontitis.

## Conclusion

nLSRH have antimicrobial activity against *Porphyromonas gingivalis* . This finding able to drive the next research to develop nLSRH as gingival and periodontitis disease is caused by *Porphyromonas gingivalis*
